# Prophylactic Intravenous Antibiotic Use in Thyroglossal Duct and Branchial Cleft Cyst Excision: A NSQIP‐P Analysis

**DOI:** 10.1002/ohn.70186

**Published:** 2026-03-03

**Authors:** Cyrus W. Abrahamson, Thomas Q. McClelland, David J. Fei‐Zhang, Jill N. D'Souza, Anthony M. Sheyn, Jeffrey C. Rastatter, Daniel C. Chelius

**Affiliations:** ^1^ Feinberg School of Medicine Northwestern University Chicago Illinois USA; ^2^ Department of Otolaryngology–Head and Neck Surgery Northwestern University Feinberg School of Medicine Chicago Illinois USA; ^3^ Department of Otolaryngology, Division of Pediatric Otolaryngology Children's Hospital of New Orleans Louisiana State University Health Sciences Center New Orleans Louisiana USA; ^4^ Department of Otolaryngology–Head and Neck Surgery University of Tennessee Health Science Center Memphis Tennessee USA; ^5^ Department of Pediatric Otolaryngology Le Bonheur Children's Hospital Memphis Tennessee USA; ^6^ Division of Pediatric Otolaryngology Ann & Robert H. Lurie Children's Hospital of Chicago Chicago Illinois USA; ^7^ Department of Otolaryngology–Head and Neck Surgery Baylor College of Medicine Houston Texas USA; ^8^ Department of Surgery, Division of Pediatric Otolaryngology Texas Children's Hospital Houston Texas USA

**Keywords:** branchial cleft cyst, pediatric otolaryngology, surgical antibiotic prophylaxis, thyroglossal duct cyst

## Abstract

**Objective:**

To identify patterns in prophylactic intravenous antibiotics (PIAB) usage for thyroglossal duct and branchial cleft cyst excisions, and to evaluate whether PIAB improve patient outcomes.

**Study Design:**

Retrospective cohort study.

**Setting:**

American College of Surgeons National Surgical Quality Improvement Program‐Pediatric (NSQIP‐P) Surgical Antibiotic Prophylaxis Database.

**Methods:**

The NSQIP‐P database was queried for pediatric patients (≤18) from 2021 to 2023 who underwent thyroglossal duct or branchial cleft cyst excision. Multivariate regressions assessed associations between PIAB use and outcomes including surgical site infection, unplanned readmission, and reoperation while adjusting for clinicodemographic factors (age, sex, race/ethnicity, admission status, surgical specialty, ASA classification, and wound classification).

**Results:**

Of 2106 TGDC and 2837 BCC patients, 83.5% and 68.0% received PIAB, respectively. Hispanic ethnicity, postoperative admission, and clean‐contaminated wound classification were associated with increased PIAB use in both groups. In BCC patients, younger age and treatment by non‐otolaryngologists predicted decreased PIAB use. While univariate analysis in TGDC cases showed lower infection rates with PIAB (OR, 0.52; 95% CI, 0.30‐0.95; *P* = .025), this was not significant on multivariate analysis (0.59, 0.34‐1.08, *P* = .074). PIAB did not significantly affect infection, readmission, or reoperation rates in either cohort on multivariate analysis.

**Conclusion:**

PIAB use in pediatric TGDC and BCC excisions varies with clinicodemographic factors but did not consistently reduce adverse outcomes. These findings underscore the need to reevaluate the necessity of PIAB and encourage further investigations to develop evidence‐based guidelines for pediatric neck mass surgery.

Thyroglossal duct cysts (TGDC) and branchial cleft cysts (BCC) are common, comprising 50% to 70% and 20% to 30% of congenital neck masses, respectively.^1^, ^2^ Considering TGDC's estimated population prevalence of ~7%, these anomalies are frequently encountered and often excised in childhood.^3^, ^4^, ^5^

While management of both anomalies is primarily surgical, the approaches vary. TGDC, typically midline near the hyoid, can be seeded hematogenously or maintain oropharyngeal connection from its embryologic origin at the tongue base.^6^, ^7^, ^8^ TGDC wound classification may be ambiguous due to suspected oropharyngeal fistula, even without obvious connection or signs of acute infection. Many otolaryngologists consider TGDC excision clean‐contaminated rather than clean; however, further studies are needed to clarify this. In contrast, BCC excisions are generally considered clean surgeries in the absence of discrete aerodigestive fistula or active infection.^9^

Perioperative antibiotics are often administered during surgical treatment of these anomalies.^10^ However, as antibiotic‐resistant bacteria are increasingly concerning, antibiotic stewardship has become a priority.^11^, ^12^ Clean and clean‐contaminated procedures face scrutiny regarding prophylactic intravenous antibiotics (PIAB) necessity and whether they provide clinically significant benefit.

To better understand PIAB administration for TGDC and BCC excisions, this study aimed to identify disparities in PIAB usage between patient populations, evaluate the effect of PIABs on postoperative outcomes, and compare PIAB usage and outcomes between the anomalies. Given limited evidence for PIAB in clean or clean‐contaminated head and neck procedures, we hypothesized PIAB administration would not be associated with a significant reduction in postoperative complications following TGDC or BCC excision. Furthermore, we anticipated PIAB use would vary across clinicodemographic factors. To test this hypothesis, we conducted a large database analysis of pediatric patients who underwent either TGDC or BCC excision.

## Methods

This retrospective cohort study follows STROBE guidelines. No IRB approval was needed as the database contains publicly available deidentified data.

### Database

This study utilized the ACS National Surgical Quality Improvement Program‐Pediatric (NSQIP‐P) which contains national datasets of patient demographics, surgical characteristics, treatment modalities, and outcomes. NSQIP‐P comprises hundreds of hospitals nationwide allowing for accurate representation of the US improving generalizability. This study utilized the Surgical Antibiotic Prophylaxis (SAP) participant use files from January 1, 2021 to December 31, 2023 which have antibiotic‐specific variables including whether patients received PIAB, antibiotics received, timing of administration, postoperative continuation, and duration of continuation.

### Inclusion/Exclusion Criteria

NSQIP‐P SAP files were queried using CPT codes for children who underwent TGDC excision (60280) or BCC excision (42810 or 42815). Patients were excluded if any of the following were present: active infection, concern for infection, or sepsis at the time of surgery (whether related or unrelated to the procedure); IV antibiotic administration before the preoperative prophylactic window (within 3 hours of procedure) for treatment of a preexisting infection; or an immunodeficiency diagnosis or cardiac history requiring PIAB. Patients with active infection or who received antibiotics before the prophylactic window were excluded as they required therapeutic not prophylactic antibiotics. Patients receiving protocolized PIAB independent of the procedure such as for immunodeficiency, immunocompromise, or cardiac risk factors were excluded because of potential bias.

The SAP dataset already filters most cases based on these criteria, but some were not excluded. Of 2238 TGDC cases identified, 69 were excluded for active infection, 9 for preoperative antibiotic administration, and 54 for immunodeficiencies or cardiac history. Of 3080 BCC cases, 127 were excluded for active infection, 9 for preoperative antibiotic administration, and 107 for immunodeficiencies or cardiac history. After exclusions, 2106 TGDC and 2837 BCC patients remained.

Notably, the NSQIP‐P SAP database provides data concerning active infection at the time of surgery but does not include historical infection data. Therefore, while patients with active infections and/or receiving IV antibiotic treatment for a recent infection were excluded, more remote infections cannot be identified. This limitation is inherent to NSQIP‐P and addressed by excluding cases with active infection or ongoing IV antibiotic therapy, ensuring antibiotic use reflected prophylaxis rather than treatment.

### Variable and Outcome Definitions

Patients were categorized by 7 factors: age, sex, race/ethnicity, admission status, surgical specialty, ASA classification, and wound classification. BCC patients had 2 additional factors included: CPT and ICD‐10. Antibiotic variables included PIAB administration, postoperative continuation, antibiotic class, and timing of administration. The list of reference variables and corresponding comparators are listed in Supplemental Table S1, available online.

Outcomes were defined using NSQIP‐P's 30‐day postoperative variables. Surgical site infection (SSI) included superficial, deep, and organ/space infections. Readmission was hospital readmission for any cause, and reoperation was any unplanned return to the operating room. All outcomes were within 30 days of surgery.

### Statistical Analysis

Multivariate models were used to analyze relationships between patient factors and outcomes. First, multivariate logistic regression assessed PIAB administration patterns across clinicodemographic groups to identify potential disparities in usage for both cohorts. Second, univariate logistic regressions assessed associations between PIAB and both clinicodemographic factors and outcomes (SSI, readmission, and reoperation). Finally, multivariate logistic regressions determined independent predictors of each outcome, including both clinicodemographic predictors and PIAB use.

Chi‐squared tests were performed within each cohort to compare clinicodemographic characteristics between patients who did and did not receive PIAB, and between cohorts among PIAB recipients. All 2 × 2 comparisons used Yates' continuity correction while comparisons larger than 2 × 2 utilized Pearson's Chi‐square test. For expected cell counts <5, Fisher's exact tests were used; these were used for ASA Class and ICD‐10 comparisons. Two‐sided *P*‐values were used for all analyses with the alpha‐criterion at 0.05. Statistical analyses were performed with R‐4.4.0.

## Results

Of 2106 TGDC and 2837 BCC patients included, 83.5% and 68.0% received PIAB, respectively. For TGDC excision, significant differences were found between those who did and did not receive PIAB for the clinicodemographic factors of admission status (*P* = .016) and wound classification (*P* = .002). SSI was significantly lower with PIAB administration, at nearly half the rate (4.9% vs 2.6%, *P* = .035). For BCC, statistically significant differences were found between those who did and did not receive PIAB based on age, race/ethnicity (*P* = .013), admission status (*P* = .009), surgical specialty, wound classification, CPT code, and ICD‐10 description (*P* = .007) (all *P* < .001 unless noted). Unlike TGDC excision, SSI was similar regardless of PIAB administration (2.2% vs 2.3%, *P* = .940) (Table 1).

**Table 1:** Patient Characteristics Stratified by Prophylactic Intravenous Antibiotic Administration

	Thyroglossal Duct Cysts			Branchial Cleft Cysts		
Characteristic	No PIAB, N = 347 (16.5%)	PIAB, N = 1759 (83.5%)	*P*	No PIAB, N = 907 (32.0%)	PIAB, N = 1930 (68.0%)	*P*
Age			.212			**<.001**
0‐2 Years	48 (21.0%)	181 (79.0%)		381 (39.4%)	587 (60.6%)	
2‐5 Years	122 (15.7%)	654 (84.3%)		254 (32.8%)	521 (67.2%)	
5‐10 Years	119 (16.8%)	591 (83.2%)		161 (27.9%)	416 (72.1%)	
10‐18 Years	58 (14.8%)	333 (85.2%)		111 (21.5%)	406 (78.5%)	
Sex			.228			.411
Male	191 (17.5%)	903 (82.5%)		434 (31.2%)	957 (68.8%)	
Female	156 (15.4%)	856 (84.6%)		473 (32.7%)	973 (67.3%)	
Race/Ethnicity			.051			**.013**
White	175 (18.3%)	780 (81.7%)		413 (34.9%)	771 (65.1%)	
Black	41 (14.5%)	241 (85.5%)		130 (28.8%)	321 (71.2%)	
Hispanic	49 (12.1%)	356 (87.9%)		137 (27.0%)	371 (73.0%)	
Asian or Pacific Islander	13 (16.0%)	68 (84.0%)		69 (33.0%)	140 (67.0%)	
Other/Unknown	69 (18.0%)	314 (82.0%)		158 (32.6%)	327 (67.4%)	
Admission Status			**.016**			**.009**
Outpatient	291 (17.5%)	1,370 (82.5%)		855 (32.6%)	1,764 (67.4%)	
Inpatient	56 (12.6%)	389 (87.4%)		52 (23.8%)	166 (76.2%)	
Surgical Specialty			.168			**<.001**
Otolaryngology	288 (17.1%)	1,400 (82.9%)		510 (27.2%)	1,362 (72.8%)	
Non‐Otolaryngology	59 (14.1%)	359 (85.9%)		397 (41.2%)	568 (58.8%)	
ASA Classification			.482			.079
ASA 1	200 (17.3%)	956 (82.7%)		551 (32.9%)	1,124 (67.1%)	
ASA 2	140 (15.6%)	755 (84.4%)		341 (30.9%)	762 (69.1%)	
ASA 3	7 (12.7%)	48 (87.3%)		12 (21.8%)	43 (78.2%)	
ASA 4	0 (0%)	0 (0%)		1 (100.0%)	0 (0.0%)	
ASA NA	0 (0%)	0 (0%)		2 (66.7%)	1 (33.3%)	
Wound Classification			**.002**			**<.001**
Clean	260 (18.2%)	1,166 (81.8%)		670 (34.7%)	1,260 (65.3%)	
Clean‐contaminated	87 (12.8%)	593 (87.2%)		237 (26.1%)	670 (73.9%)	
CPT Code			NA			**<.001**
42810	NA	NA		572 (37.1%)	971 (62.9%)	
42815	NA	NA		335 (25.9%)	959 (74.1%)	
ICD‐10 Description			NA			**.007**
Q18.0	NA	NA		535 (33.1%)	1,081 (66.9%)	
Q17.0/18.1	NA	NA		198 (28.7%)	493 (71.3%)	
R22.1	NA	NA		26 (22.4%)	90 (77.6%)	
Q18.2	NA	NA		148 (35.7%)	266 (64.3%)	
Surgical Site Infection			**.035**			.940
No Infection	330 (95.1%)	1,713 (97.4%)		887 (97.8%)	1,885 (97.7%)	
Surgical Site Infection	17 (4.9%)	46 (2.6%)		20 (2.2%)	45 (2.3%)	
Readmission			.376			.344
Not Readmitted	339 (97.7%)	1,733 (98.5%)		900 (99.2%)	1,922 (99.6%)	
Readmission	8 (2.3%)	26 (1.5%)		7 (0.8%)	8 (0.4%)	
Reoperation			.857			.989
No Reoperation	340 (98.0%)	1,729 (98.3%)		902 (99.4%)	1,921 (99.5%)	
Reoperation	7 (2.0%)	30 (1.7%)		5 (0.6%)	9 (0.5%)	

Percentages calculated horizontally within groups for demographic factors and calculated vertically by PIAB administration for outcomes. *P*‐values in bold indicate statistical significance.

Analysis between TGDC and BCC patients who received PIAB revealed differences based on age, race/ethnicity, admission status, and surgical specialty (all *P* ≤ .001). Differences in postoperative outcomes were also found for readmission and reoperation (both *P* < .001), with TGDC patients having increased rates of these events compared with BCC patients when both receive PIAB (Supplemental Table S2, available online). Supplemental univariate logistic regressions detailing associations between clinicodemographic factors and PIAB administration are provided in Supplemental Tables S3 and S4, available online.

### Prophylactic Intravenous Antibiotic Administration

On multivariate logistic regression, several factors were associated with PIAB administration. For both cohorts, Hispanic ethnicity was associated with ~50% higher odds of PIAB (TGDC: OR, 1.56; 95% CI, 1.12‐2.22; *P* = .011 | BCC: OR, 1.49; 95% CI, 1.18‐1.90; *P* = .001) and clean‐contaminated wound classification was linked to a 30% to 40% increase in PIAB administration (TGDC: 1.43, 1.10‐1.88, *P* = .008 | BCC: 1.29, 1.07‐1.55, *P* = .007). Additionally, TGDC patients admitted inpatient (1.57, 1.14‐2.19, *P* = .007) were ~60% more likely to receive PIAB compared to those outpatient. BCC patients aged 0 to 2 (0.54, 0.42‐0.70, *P* < .001) and 2 to 5 (0.64, 0.49‐0.83, *P* < .001) had 35% to 45% lower odds of PIAB administration compared with those aged 10 to 18, and non‐otolaryngologists (0.70, 0.58‐0.84, *P* < .001) were 30% less likely to use PIAB versus otolaryngologists. Patients with BCC extending beneath subcutaneous tissue or into the pharynx (CPT 42815) had ~50% higher odds of PIAB versus those whose BCC were confined to skin and soft tissue (CPT 42810) (1.54, 1.29‐1.82, *P* < .001) (Table 2).

**Table 2:** Multivariate Logistic Regression of Prophylactic Intravenous Antibiotic Administration

	Thyroglossal Duct Cyst			Branchial Cleft Cyst		
Characteristic	OR	95% CI	*P*‐value	OR	95% CI	*P*‐value
Age (0‐2 years)	0.68	0.45, 1.05	.083	0.54	0.42, 0.70	**<.001**
Age (2‐5 years)	0.97	0.68, 1.36	.842	0.64	0.49, 0.83	**<.001**
Age (5‐10 years)	0.89	0.63, 1.25	.510	0.76	0.57, 1.01	.061
Sex – Female	1.16	0.92, 1.47	.211	0.89	0.76, 1.05	.163
Race/Ethnicity – Black	1.32	0.92, 1.93	.147	1.27	1.00, 1.63	.055
Race/Ethnicity – Hispanic	1.56	1.12, 2.22	**.011**	1.49	1.18, 1.90	**.001**
Race/Ethnicity – Asian	1.06	0.58, 2.06	.859	0.98	0.71, 1.36	.885
Admission Status – Inpatient	1.57	1.14, 2.19	**.007**	1.35	0.96, 1.93	.089
Surgical Specialty – Non‐Otolaryngology	1.30	0.96, 1.79	.091	0.70	0.58, 0.84	**<.001**
ASA Class – ASA 2+	1.12	0.89, 1.42	.341	0.99	0.84, 1.17	.894
Wound Class – Clean‐contaminated	1.43	1.10, 1.88	**.008**	1.29	1.07, 1.55	**.007**
CPT Code – 42815	NA	NA	NA	1.54	1.29, 1.82	**<.001**
ICD – Auricular (Q17.0/18.1)	NA	NA	NA	1.11	0.90, 1.38	.332
ICD – Swelling, Mass, Lump in Neck (R22.1)	NA	NA	NA	1.43	0.92, 2.30	.126
ICD – Other (Q18.2)	NA	NA	NA	0.99	0.78, 1.25	.901

Abbreviations: CI, confidence interval; OR, odds ratio. *P*‐values in bold indicate statistical significance.

Postoperative PIAB continuation was also evaluated for both anomalies. Inpatient admission had a fourfold increase in odds of postoperative PIAB continuation (TGDC: 4.15, 3.02‐5.70, *P* < .001 | BCC: 4.32, 2.37‐7.79, *P* < .001). Nonotolaryngologists were 50% to 85% less likely to continue postoperative PIAB than otolaryngologists (TGDC: 0.50, 0.31‐0.77, *P* = .002 | BCC: 0.13, 0.03‐0.36, *P* < .001). BCC excisions with ICD codes indicating auricular abnormalities had a 95% lower likelihood of postoperative continuation versus those with sinus, fistula, or cyst of branchial cleft (0.04, 0.00‐0.19, *P* = .002) (Supplemental Table S5, available online).

### Postoperative Infection

Regarding PIAB administration's effect on outcomes, on univariate analysis, PIAB was associated with ~50% lower odds of SSI in TGDC patients (0.52, 0.30‐0.95, *P* = .025). PIAB were not associated with any change to SSI for BCC patients (1.06, 0.63‐1.84, *P* = .834). Neither TGDC nor BCC patients had any difference in odds of readmission or reoperation based on PIAB (Table 3).

**Table 3:** Univariate Logistic Regression of Prophylactic Intravenous Antibiotic Administration on Outcomes

	Thyroglossal Duct Cyst			Branchial Cleft Cyst		
Outcome	OR	95% CI	*P*‐value	OR	95% CI	*P*‐value
Wound Infection	0.52	0.30, 0.95	**.025**	1.06	0.63, 1.84	.834
Readmission	0.64	0.30, 1.51	.268	0.54	0.19, 1.53	.228
Reoperation	0.84	0.39, 2.10	.687	0.85	0.29, 2.76	.764

Abbreviations: CI, confidence interval; OR, odds ratio. *P*‐values in bold indicate statistical significance.

Multivariate analysis on SSI revealed several findings. While PIAB administration demonstrated a similar 40% reduction in odds of SSI for TGDC patients on multivariate analysis, it was not statistically significant (0.59, 0.34‐1.08, *P* = .074). For TGDC patients, ages 0 to 2 (10.8, 2.93‐69.9, *P* = .002), 2 to 5 (6.61, 1.96‐41.2, *P* = .010), and 5 to 10 (5.62, 1.63‐35.3, *P* = .020) were associated with a 5‐11‐fold increased SSI risk compared to patients 10‐18, with SSI risk decreasing as patient age increased. Additionally, Hispanic patients had ~70% lower odds of postoperative infection (0.27, 0.08‐0.67, *P* = .013). BCC excision revealed its own distinct findings, with those admitted inpatient having 2.6‐fold higher odds of SSI (2.61, 1.22‐5.25, *P* = .009). Interestingly, patients cared for by nonotolaryngologists had ~60% decreased odds of SSI (0.41, 0.18‐0.83, *P* = .020). BCC patients with auricular abnormalities had more than twice the odds of SSI versus those with sinus, fistula, or cyst of branchial cleft (2.15, 1.20‐3.86, *P* = .010) (Table 4). Among BCC patients who received PIAB, patients who received clindamycin had fourfold higher odds of SSI compared with those who received a cephalosporin (3.86, 1.10‐10.6, *P* = .017) (Supplemental Table S6, available online).

**Table 4:** Multivariate Logistic Regression of Postoperative Wound Infection

	Thyroglossal Duct Cyst			Branchial Cleft Cyst		
Characteristic	OR	95% CI	*P*‐value	OR	95% CI	*P*‐value
Age (0‐2 years)	10.8	2.93, 69.9	**.002**	1.77	0.81, 4.09	.163
Age (2‐5 years)	6.61	1.96, 41.2	**.010**	1.42	0.65, 3.28	.391
Age (5‐10 years)	5.62	1.63, 35.3	**.020**	1.74	0.80, 3.98	.173
Sex – Female	0.72	0.42, 1.20	.210	1.33	0.81, 2.23	.265
Race/Ethnicity – Black	0.51	0.19, 1.15	.138	1.04	0.46, 2.22	.925
Race/Ethnicity – Hispanic	0.27	0.08, 0.67	**.013**	1.05	0.47, 2.22	.894
Race/Ethnicity – Asian	0.63	0.10, 2.17	.533	1.23	0.44, 3.00	.665
Admission Status – Inpatient	1.26	0.66, 2.28	.469	2.61	1.22, 5.25	**.009**
Surgical Specialty – Non‐Otolaryngology	0.62	0.27, 1.26	.223	0.41	0.18, 0.83	**.020**
ASA Class – ASA 2+	1.26	0.76, 2.11	.369	1.23	0.73, 2.04	.434
Wound Class – Clean‐contaminated	0.81	0.44, 1.42	.479	1.45	0.87, 2.41	.153
Antibiotic Prophylaxis	0.59	0.34, 1.08	.074	0.91	0.53, 1.60	.734
CPT Code – 42815	NA	NA	NA	1.12	0.66, 1.89	.685
ICD – Auricular (Q17.0/18.1)	NA	NA	NA	2.15	1.20, 3.86	**.010**
ICD – Swelling, Mass, Lump in Neck (R22.1)	NA	NA	NA	0.38	0.02, 1.82	.345
ICD – Other (Q18.2)	NA	NA	NA	0.68	0.23, 1.64	.439

Abbreviations: CI, confidence interval; OR, odds ratio. *P*‐values in bold indicate statistical significance.

### Readmission and Reoperation

Regarding readmission and reoperation, TGDC patients ages 0‐2 had borderline significant 3.4‐fold higher odds of readmission within 30 days (3.41, 1.05‐13.0, *P* = .049) and 4.9‐fold higher odds of reoperation within 30 days (4.94, 1.40‐22.9, *P* = .020) compared with patients ages 10 to 18. BCC patients admitted inpatient had nearly 6‐fold higher odds of readmission within 30 days (5.86, 1.54‐20.3, *P* = .006) and over 4‐fold higher odds of reoperation within 30 days (4.27, 1.00‐15.7, *P* = .036). Finally, Hispanic BCC patients (4.09, 1.03‐17.3, *P* = .043) had 4‐fold increased odds of readmission within 30 days (Tables 5 and 6). No differences in outcomes were found based on postoperative continuation, antibiotic class, or timing of administration (Supplemental Tables S7 and S8, available online).

**Table 5:** Multivariate Logistic Regression of Readmission Within 30 Days

	Thyroglossal Duct Cyst			Branchial Cleft Cyst		
Characteristic	OR	95% CI	*P*‐value	OR	95% CI	*P*‐value
Age (0‐2 Years)	3.41	1.05, 13.0	**.049**	1.17	0.24, 6.36	.843
Age (2‐5 Years)	1.89	0.68, 6.69	.263	1.29	0.30, 6.54	.732
Age (5‐10 Years)	0.93	0.28, 3.60	.913	1.10	0.20, 6.12	.913
Sex – Female	0.80	0.39, 1.59	.531	0.87	0.30, 2.47	.785
Race/Ethnicity – Black	0.53	0.12, 1.59	.313	1.61	0.22, 8.59	.588
Race/Ethnicity – Hispanic	0.83	0.30, 2.01	.696	4.09	1.03, 17.3	**.043**
Race/Ethnicity – Asian	0.65	0.04, 3.36	.685	0.00	0.00, Inf	.990
Admission Status – Inpatient	1.13	0.46, 2.51	.777	5.86	1.54, 20.3	**.006**
Surgical Specialty – Non‐Otolaryngology	0.56	0.17, 1.46	.290	0.36	0.05, 1.51	.209
ASA Class – ASA 2+	1.23	0.62, 2.47	.551	2.59	0.89, 8.58	.092
Wound Class – Clean‐contaminated	1.05	0.48, 2.15	.899	1.69	0.57, 4.94	.329
Antibiotic Prophylaxis	0.68	0.32, 1.64	.356	0.42	0.14, 1.25	.109
CPT Code – 42815	NA	NA	NA	1.08	0.35, 3.35	.894
ICD – Auricular (Q17.0/18.1)	NA	NA	NA	0.56	0.12, 2.09	.424
ICD – Swelling, Mass, Lump in Neck (R22.1)	NA	NA	NA	0.00	0.00, Inf	.992
ICD – Other (Q18.2)	NA	NA	NA	0.43	0.02, 2.28	.420

Abbreviations: CI, confidence interval; OR, odds ratio. *P*‐values in bold indicate statistical significance.

**Table 6:** Multivariate Logistic Regression of Reoperation Within 30 Days

	Thyroglossal Duct Cyst			Branchial Cleft Cyst		
Characteristic	OR	95% CI	*P*‐value	OR	95% CI	*P*‐value
Age (0‐2 Years)	4.94	1.40, 22.9	**.020**	0.82	0.14, 4.73	.814
Age (2‐5 Years)	2.29	0.73, 10.1	.199	1.28	0.30, 6.45	.742
Age (5‐10 Years)	2.50	0.80, 11.0	.156	0.92	0.17, 5.09	.920
Sex – Female	0.87	0.44, 1.68	.677	0.51	0.16, 1.51	.237
Race/Ethnicity – Black	0.16	0.01, 0.79	.077	1.66	0.21, 10.3	.586
Race/Ethnicity – Hispanic	1.23	0.54, 2.61	.603	3.11	0.65, 16.6	.152
Race/Ethnicity – Asian	0.51	0.03, 2.59	.521	1.74	0.08, 14.4	.637
Admission Status – Inpatient	1.81	0.82, 3.74	.123	4.27	1.00, 15.7	**.036**
Surgical Specialty – Non‐Otolaryngology	0.73	0.24, 1.74	.514	0.42	0.06, 1.80	.295
ASA Class – ASA 2 +	1.08	0.55, 2.09	.828	1.39	0.46, 4.19	.554
Wound Class – Clean‐contaminated	0.64	0.28, 1.33	.257	1.71	0.57, 5.19	.332
Antibiotic Prophylaxis	0.90	0.41, 2.26	.798	0.67	0.22, 2.26	.491
CPT Code – 42815	NA	NA	NA	0.84	0.26, 2.61	.766
ICD – Auricular (Q17.0/18.1)	NA	NA	NA	1.16	0.31, 3.99	.819
ICD – Swelling, Mass, Lump in Neck (R22.1)	NA	NA	NA	0.00	0.00, Inf	.988
ICD – Other (Q18.2)	NA	NA	NA	0.57	0.03, 3.23	.601

Abbreviations: CI, confidence interval; OR, odds ratio. *P*‐values in bold indicate statistical significance.

## Discussion

TGDC and BCC are common pediatric head and neck masses requiring surgical management. Despite their routine nature, complications can occur, albeit rarely.^13^, ^14^, ^15^ To address infectious complications, antibiotics are often used; however, practices surrounding PIAB vary significantly. Given growing emphasis on antibiotic stewardship and limited evidence of benefit of PIAB for other clean or clean‐contaminated head and neck procedures, we hypothesized that PIAB do not meaningfully reduce postoperative complications. Therefore, this study is the first to identify PIAB disparities in PIAB usage for TGDC and BCC excisions, better understand PIABs effects on outcomes for TGDC and BCC excisions, and compare PIAB usage and outcomes between TGDC and BCC.

Our analysis revealed similarities in PIAB administration between TGDC and BCC. For both, inpatient admission and clean‐contaminated wounds were more likely to receive PIAB. This is expected as these patients likely have more complicated conditions than those undergoing outpatient procedures and with clean wounds. Additionally, Hispanic patients had increased odds of PIAB, possibly reflecting heightened awareness of prior studies on race/ethnicity disparities in care.^13^, ^14^

This is where the similarities in PIAB administration end. While TGDC had no differences in PIAB administration based on age, younger BCC patients had decreased odds of PIAB administration. Considering that BCC excision is considered a clean surgery, and that we observed no differences in odds of complications by age for BCC, one hypothesis is that surgeons may avoid PIAB in younger patients to avoid side effects, consistent with some prior literature on PIAB use in children.^16^ Another possible explanation is that BCC in teenagers are often recognized during infection, biasing surgeons toward PIAB, even during delayed surgery without obvious infection. Interestingly, PIAB usage was more common in patients managed by otolaryngologists. While this may reflect underlying referral pattens based on factors like prior infection, otolaryngologists have been cataloged using antibiotics more liberally than other specialties before.^17^, ^18^ This emphasizes the need to revisit evidence‐based guidelines and ensure adherence. Finally, BCC with extension beneath subcutaneous tissue or into pharynx (CPT 42815) had increased PIAB odds, reflecting potentially higher infection risk. However, prior investigations found no significant differences in complications between CPT codes.^14^, ^19^ These nuances, while unique to BCC, require further investigation.

Despite few PIAB administration differences for TGDC, several efficacy findings emerged. Univariate analysis showed decreased odds of SSI with PIABs, and while multivariate analysis demonstrated a similar magnitude of OR reduction, it was not statistically significant. Prior studies have questioned PIAB usage for TGDC, and while this work suggests that PIAB may be beneficial, further research must clarify the exact effect on SSI.^20^, ^21^ PIAB administration had no effect on readmission or reoperation within 30 days. These findings suggest that while PIAB may reduce SSI risk for TGDC excisions, they may not reduce risk substantially enough to prevent more severe outcomes.

While PIAB were shown to potentially reduce SSI for TGDC, PIAB use was not associated with reduced SSI risk for BCC. Similarly, PIAB were not shown to affect readmission or reoperation within 30 days. These results indicate no clear benefit of PIAB for BCC patients. While PIAB showed no benefit for BCC, we observed notable findings expanding upon and affirming prior work. Our study found no outcome differences between CPT codes, supporting Moroco et al.'s hypothesis that CPT code is not a sufficient marker of infectious risk.^14^, ^19^ Since CPT may not be an adequate measure of complexity, we theorized that location could be relevant. BCC with auricular abnormalities had increased odds of infection, potentially reflecting that preauricular BCC are typically after there has been an infectious complication. While auricular anomalies are not true BCC, they were included because prior studies have defined BCC using CPT alone, and we wanted to assess if this introduced confounding.^4^, ^14^, ^19^ By utilizing multivariate analyses, we better controlled for confounding and isolated the effect of location, casting doubt on using location alone as a marker of infectious risk.

Neither postoperative continuation nor timing of PIAB administration impacted outcomes among TGDC or BCC patients. Additionally, antibiotic choice had minimal effect, with only BCC patients receiving clindamycin having increased odds of SSI compared to those receiving a cephalosporin. Overall, these findings support prior investigations indicating that specifics on PIAB timing or postoperative continuation may have little effect on outcomes.^22^, ^23^, ^24^ The fact that specifics matter little supports the idea that PIAB may not provide clinical benefit.

Comparing TGDC and BCC outcomes allows us to better understand the role for PIAB for each. As shown, otolaryngologists have wide‐ranging preferences in PIAB use between TGDC and BCC excisions. While differences were seen in outcomes between the two procedures, statistical significance is not synonymous with clinical significance. The two procedures have remarkably low complication rates, and while TGDC excision saw possible efficacy for PIAB, BCC saw no benefit. Despite this, most patients undergoing each surgery received PIAB. While antibiotics will always be needed for active infection, immunocompromised patients, or cardiac prophylaxis, without these or other infectious signs, PIAB may not be necessary. For clean or clean‐contaminated otolaryngology surgeries, prior studies have demonstrated that PIAB do not provide clinical benefit, and this study supports this conclusion for these procedures.^10^, ^25^, ^26^

While this work has provided insight into PIAB use, it has limitations, many related to NSQIP‐P. While NSQIP‐P is an invaluable resource, it is subject to the limitations of a retrospective database. First, while we evaluated PIAB use, NSQIP‐P does not capture information on oral antibiotics, preventing us from accounting for concurrent or postoperative oral antibiotic use. This represents a significant potential source of confounding, as oral antibiotics are often used and may independently reduce infection risk. If patients who did not receive PIAB were more likely to receive oral antibiotics, the benefit of PIAB could be underestimated, and, conversely, if patients who received PIAB were more likely to also receive oral antibiotics, the benefit could be overestimated. Second, though de‐identification improves generalizability, it obscures individual surgeons' or institutions' preferences and protocols. These variables are poorly captured by a database and contribute to the potential for unmeasured confounding inherent to all database studies. Although we identified factors such as surgeon preferences, numerous unmeasured variables could influence both PIAB administration and infection risk. For example, a surgeon's concern about a case may be influenced by unmeasured factors not well captured by the database influencing the likelihood of PIAB administration and infection risk, thereby clouding the true effect of PIAB on SSI. Third, many of the same factors that make surgeons more likely to give PIAB, like age and inpatient admission, also increase infection risk. Because these overlap, it can be difficult for regression models to fully separate their effects. As a result, what may appear as overuse of PIAB could reflect appropriately increased use in higher‐risk patients. Moreover, within smaller high‐risk subgroups, like inpatient cases, higher PIAB use may have reduced an even greater underlying infection risk, potentially making the observed benefit appear smaller than it truly is. Finally, while most complications addressed by antibiotics occur within 30 days, NSQIP‐P's 30‐day limit means we may have missed indolent or late infections. Future work should examine whether PIAB prevents longer‐term complications. Considering these limitations, individuals and institutions may have their own unique considerations for PIAB usage and should evaluate PIAB use within the context of their environment and patient population.

Notably, the NSQIP‐P sampling strategy may influence case inclusion. In high‐volume surgical centers, not every case is captured; instead, cases are sampled in an 8‐day cycle.^27^ Within each cycle, the first 35 consecutive cases meeting inclusion/exclusion criteria are included, and 40 of the 46 annual 8‐day cycles are submitted. Although the 8‐day rotation aims to give all days an equal chance of inclusion, it is not perfect and could introduce bias. While it is unlikely that TGDC or BCC occur frequently enough even at large hospitals for sampling to substantially alter inclusion, the possibility remains.

Another consideration is that reliance on ICD codes may introduce additional uncertainty. Since NSQIP‐P collects cases by CPT, only patients with the specified CPT code who also have the ICD code of interest are captured. Some relevant cases could be missed, or cases could be misclassified, potentially biasing to the findings. For example, some BCC excisions may be coded as generic deep neck mass excisions or neck dissections, and conversely, cases coded as auricular anomalies may not actually represent BCC excisions. Despite this limitation, including ICD codes allows for exploratory assessment of location‐specific factors, but results should be interpreted with caution and validated in future studies.

This study is the first to investigate PIAB usage for TGDC and BCC excisions in a large population and lays the foundation for future investigations. One area for further exploration is prospective evaluation of PIAB for reducing SSIs after TGDC excision. By better understanding this relationship, we can improve antibiotic stewardship while optimizing outcomes. Another area for study is into surgical classification. While BCC excision is often considered clean because of its low infection risk, TGDC excision has more debate surrounding it. These results present evidence that TGDC excision should be considered a clean surgery and that, while not as clean as BCC excision, it still has low infection risk. The final avenue for investigation is into BCC classification as little work has investigated outcomes and management of BCC based on depth and location. Therefore, research into these anomalies is necessary so surgeons can tailor management for each patient.

## Conclusion

TGDC and BCC excision are among the most common pediatric otolaryngology procedures, yet PIAB use varies widely. PIAB use, while showing reduced SSI for TGDC on univariate analysis, did not demonstrate benefit on multivariate analysis nor did it reduce odds of postoperative readmission or reoperation. PIAB demonstrated no benefit on univariate or multivariate analysis for BCC. These findings illustrate the need for otolaryngologists to revisit perioperative strategies for these procedures and clean and clean‐contaminated procedures generally.

## Author Contributions

**Cyrus W. Abrahamson**, conceptualization, data curation, formal analysis, methodology, writing–original draft; **Thomas Q. McClelland**, formal analysis, methodology, writing–original draft; **David J. Fei‐Zhang**, methodology, writing–review and editing; **Jill N. D'Souza**, conceptualization, supervision, writing–review and editing; **Anthony M. Sheyn**, supervision, writing–review and editing; **Jeffrey C. Rastatter**, resources, supervision, writing–review and editing; **Daniel C. Chelius**, conceptualization, methodology, project administration, supervision, writing–review and editing.

## Disclosures

### Competing interests

Dr Chelius has received a stipend from the American‐Academy of Otolaryngology outside the submitted work. No other current or potential conflicts of interest were reported.

### Funding source

None.

## Supporting information

Supp_TableS1.docx.

Supp_TableS2.docx.

Supp_TablesS3‐4.docx.

Supp_TableS5.docx.

Supp_TableS6‐8.docx.
