# Supplementary material for: Prophylactic Intravenous Antibiotic Use in Thyroglossal Duct and Branchial Cleft Cyst Excision: A NSQIP‐P Analysis
Source: Otolaryngol Head Neck Surg. 2026 Mar 3;174(5):1243–52. doi: 10.1002/ohn.70186 (PMC13126438; doi:10.1002/ohn.70186)
Supplement: Supplementary file 1 — Supp_TableS1.docx. [file OHN-174-1243-s003.docx]

| **Variable/Outcome** | **Reference** | **Comparator(s)** |
| --- | --- | --- |
| **Age** | 10-18 years | 0-2 years  2-5 years  5-10 years |
| **Sex** | Male | Female |
| **Race/Ethnicity** | White | Black  Hispanic  Asian or Pacific Islander  Other/Unknown |
| **Admission Status** | Outpatient | Inpatient |
| **Surgical Specialty** | Otolaryngology | Non-otolaryngology |
| **ASA Classification** | ASA I | ASA II+ |
| **Wound Classification** | Clean | Clean-contaminated |
| **CPT (BCC only)** | 42810 (confined to skin and soft tissue) | 42815 (extension beneath subcutaneous tissue or into the pharynx) |
| **ICD-10 (BCC only)** | Q18.0 (sinus, fistula, or cyst of branchial cleft) | Q17.0/Q18.1 (accessory auricle or preauricular sinus/cyst)  R22.1 (localized swelling/mass/lump, neck)  Q18.2 (other branchial cleft malformations) |
| **PIAB Administration** | No | Yes |
| **Postoperative Continuation** | No | Yes |
| **Antibiotic Class** | Cephalosporin | Penicillin  Clindamycin  Other |
| **Timing of Administration** | During the operation | 0-5 min prior  5-10 min prior  10-15 min prior  15-30 min prior  30+ min prior |

**Table S1:** Reference Groups and Corresponding Comparators for Variables of Interest.
