# Supplementary material for: Prophylactic Intravenous Antibiotic Use in Thyroglossal Duct and Branchial Cleft Cyst Excision: A NSQIP‐P Analysis
Source: Otolaryngol Head Neck Surg. 2026 Mar 3;174(5):1243–52. doi: 10.1002/ohn.70186 (PMC13126438; doi:10.1002/ohn.70186)
Supplement: Supplementary file 2 — Supp_TableS2.docx. [file OHN-174-1243-s005.docx]

| **Characteristic** | **Thyroglossal Duct Cyst** | **Branchial Cleft Cyst** | **p** |
| --- | --- | --- | --- |
| **Age** |  |  | **<0.001** |
| 0-2 Years | 181 (10.3%) | 587 (**30.4**%) |  |
| 2-5 Years | 654 (37.2%) | 521 (27.0%) |  |
| 5-10 Years | 591 (33.6%) | 416 (21.6%) |  |
| 10-18 Years | 333 (18.9%) | 406 (21.0%) |  |
| **Sex** |  |  | 0.303 |
| Male | 903 (51.3%) | 957 (49.6%) |  |
| Female | 856 (48.7%) | 973 (50.4%) |  |
| **Race/Ethnicity** |  |  | **<0.001** |
| White | 780 (44.3%) | 771 (39.9%) |  |
| Black | 241 (13.7%) | 321 (16.6%) |  |
| Hispanic | 356 (20.2%) | 371 (19.2%) |  |
| Asian or Pacific Islander | 68 (3.9%) | 140 (7.3%) |  |
| Other/Unknown | 314 (17.9%) | 327 (16.9%) |  |
| **Admission Status** |  |  | **<0.001** |
| Outpatient | 1,370 (77.9%) | 1,764 (91.4%) |  |
| Inpatient | 389 (22.1%) | 166 (8.6%) |  |
| **Surgical Specialty** |  |  | **<0.001** |
| Otolaryngology | 1,400 (79.6%) | 1,362 (70.6%) |  |
| Non-Otolaryngology | 359 (20.4%) | 568 (29.4%) |  |
| **ASA Classification** |  |  | 0.051 |
| ASA 1 | 956 (54.4%) | 1,124 (58.2%) |  |
| ASA 2 | 755 (42.9%) | 762 (39.5%) |  |
| ASA 3 | 48 (2.7%) | 43 (2.2%) |  |
| ASA 4 | 0 (0%) | 0 (0.0%) |  |
| ASA NA | 0 (0%) | 1 (0.1%) |  |
| **Wound Classification** |  |  | 0.544 |
| Clean | 1,166 (66.3%) | 1,260 (65.3%) |  |
| Clean/Contaminated | 593 (33.7%) | 670 (34.7%) |  |
| **Surgical Site Infection** |  |  | 0.654 |
| No Infection | 1,713 (97.4%) | 1,885 (97.7%) |  |
| Surgical Site Infection | 46 (2.6%) | 45 (2.3%) |  |
| **Readmission** |  |  | **0.001** |
| Not Readmitted | 1,733 (98.5%) | 1,922 (99.6%) |  |
| Readmission | 26 (1.5%) | 8 (0.4%) |  |
| **Reoperation** |  |  | **<0.001** |
| No Reoperation | 1,729 (98.3%) | 1,921 (99.5%) |  |
| Reoperation | 30 (1.7%) | 9 (0.5%) |  |

**Table S2:** Comparison of clinicodemographic characteristics and outcomes by surgery type among patients who received prophylactic intravenous antibiotics.
