# Supplementary material for: Prophylactic Intravenous Antibiotic Use in Thyroglossal Duct and Branchial Cleft Cyst Excision: A NSQIP‐P Analysis
Source: Otolaryngol Head Neck Surg. 2026 Mar 3;174(5):1243–52. doi: 10.1002/ohn.70186 (PMC13126438; doi:10.1002/ohn.70186)
Supplement: Supplementary file 3 — Supp_TablesS3‐4.docx. [file OHN-174-1243-s002.docx]

|  | **Thyroglossal Duct Cyst** | | | **Branchial Cleft Cyst** | | |
| --- | --- | --- | --- | --- | --- | --- |
| **Characteristic** | **OR** | **95% CI** | **p-value** | **OR** | **95% CI** | **p-value** |
| **Age (0-2 Years)** | 0.66 | 0.43, 1.01 | 0.051 | 0.42 | 0.33, 0.54 | **<0.001** |
| **Age (2-5 Years)** | 0.93 | 0.66, 1.31 | 0.692 | 0.56 | 0.43, 0.72 | **<0.001** |
| **Age (5-10 Years)** | 0.87 | 0.61, 1.21 | 0.405 | 0.71 | 0.53, 0.93 | **0.014** |
| **Sex – Female** | 1.16 | 0.92, 1.46 | 0.207 | 0.93 | 0.80, 1.09 | 0.389 |
| **Race/Ethnicity – Black** | 1.32 | 0.92, 1.93 | 0.142 | 1.32 | 1.05, 1.68 | **0.020** |
| **Race/Ethnicity – Hispanic** | 1.63 | 1.17, 2.31 | **0.005** | 1.45 | 1.16, 1.83 | **0.001** |
| **Race/Ethnicity – Asian** | 1.17 | 0.65, 2.27 | 0.610 | 1.09 | 0.80, 1.49 | 0.601 |
| **Admission Status – Inpatient** | 1.48 | 1.09, 2.02 | **0.013** | 1.55 | 1.13, 2.15 | **0.008** |
| **Surgical Specialty –  Non-Otolaryngology** | 1.25 | 0.93, 1.71 | 0.147 | 0.54 | 0.45, 0.63 | **<0.001** |
| **ASA Class – ASA 2+** | 1.14 | 0.91, 1.44 | 0.261 | 1.11 | 0.95, 1.31 | 0.187 |
| **Wound Class – Clean/Contaminated** | 1.52 | 1.17, 1.98 | **0.002** | 1.50 | 1.26, 1.79 | **<0.001** |
| **CPT Code – 42815** | NA | NA | NA | 1.69 | 1.44, 1.98 | **<0.001** |
| **ICD – Auricular (Q17.0/18.1)** | NA | NA | NA | 1.23 | 1.02, 1.50 | **0.036** |
| **ICD – Swelling, Mass, Lump in Neck (R22.1)** | NA | NA | NA | 1.71 | 1.11, 2.73 | **0.019** |
| **ICD – Other (Q18.2)** | NA | NA | NA | 0.89 | 0.71, 1.12 | 0.310 |

**Table S3.** Univariate logistic regression of prophylactic intravenous antibiotic administration.

| **Characteristic** | **OR** | **95% CI** | **p-value** |
| --- | --- | --- | --- |
| **Age (0-2 Years)** | 2.66 | 2.13, 3.32 | **<0.001** |
| **Age (2-5 Years)** | 0.65 | 0.54, 0.79 | **<0.001** |
| **Age (5-10 Years)** | 0.58 | 0.48, 0.70 | **<0.001** |
| **Sex – Female** | 1.07 | 0.94, 1.22 | 0.288 |
| **Race/Ethnicity – Black** | 1.35 | 1.11, 1.64 | **0.003** |
| **Race/Ethnicity – Hispanic** | 1.63 | 1.17, 2.31 | 0.556 |
| **Race/Ethnicity – Asian** | 1.17 | 0.65, 2.27 | **<0.001** |
| **Admission Status – Inpatient** | 0.33 | 0.27, 0.40 | **<0.001** |
| **Surgical Specialty –  Non-Otolaryngology** | 1.63 | 1.40, 1.89 | **<0.001** |
| **ASA Class – ASA 2+** | 0.85 | 0.75, 0.97 | **0.017** |
| **Wound Class – Clean/Contaminated** | 1.05 | 0.91, 1.20 | 0.521 |
| **Wound Infection** | 0.89 | 0.59, 1.35 | 0.579 |
| **Readmission** | 0.28 | 0.13, 0.61 | **<0.001** |
| **Reoperation** | 0.27 | 0.13, 0.57 | **<0.001** |

**Table S4.** Univariate logistic regression comparing BCC patient characteristics and outcomes to TGDC patients.
