# Supplementary material for: Prophylactic Intravenous Antibiotic Use in Thyroglossal Duct and Branchial Cleft Cyst Excision: A NSQIP‐P Analysis
Source: Otolaryngol Head Neck Surg. 2026 Mar 3;174(5):1243–52. doi: 10.1002/ohn.70186 (PMC13126438; doi:10.1002/ohn.70186)
Supplement: Supplementary file 4 — Supp_TableS5.docx. [file OHN-174-1243-s001.docx]

|  | **Thyroglossal Duct Cyst** | | | **Branchial Cleft Cyst** | | |
| --- | --- | --- | --- | --- | --- | --- |
| **Characteristic** | **OR** | **95% CI** | **p-value** | **OR** | **95% CI** | **p-value** |
| **Age (0-2 Years)** | 1.18 | 0.68, 2.02 | 0.553 | 0.51 | 0.25, 1.02 | 0.061 |
| **Age (2-5 Years)** | 0.70 | 0.45, 1.08 | 0.101 | 0.55 | 0.28, 1.05 | 0.074 |
| **Age (5-10 Years)** | 1.25 | 0.84, 1.90 | 0.278 | 0.58 | 0.28, 1.14 | 0.123 |
| **Sex – Female** | 0.79 | 0.58, 1.06 | 0.112 | 1.40 | 0.85, 2.33 | 0.191 |
| **Race/Ethnicity – Black** | 0.93 | 0.56, 1.49 | 0.768 | 0.52 | 0.19, 1.21 | 0.163 |
| **Race/Ethnicity – Hispanic** | 1.16 | 0.79, 1.70 | 0.435 | 0.89 | 0.39, 1.85 | 0.760 |
| **Race/Ethnicity – Asian** | 0.91 | 0.42, 1.81 | 0.804 | 0.43 | 0.10, 1.32 | 0.192 |
| **Admission Status – Inpatient** | 4.15 | 3.02, 5.70 | **<0.001** | 4.32 | 2.37, 7.79 | **<0.001** |
| **Surgical Specialty –  Non-Otolaryngology** | 0.50 | 0.31, 0.77 | **0.002** | 0.13 | 0.03, 0.36 | **<0.001** |
| **ASA Class – ASA 2+** | 0.97 | 0.71, 1.30 | 0.817 | 1.30 | 0.79, 2.15 | 0.298 |
| **Wound Class – Clean/Contaminated** | 1.14 | 0.84, 1.55 | 0.399 | 1.37 | 0.83, 2.27 | 0.213 |
| **CPT Code – 42815** | NA | NA | NA | 1.40 | 0.81, 2.49 | 0.238 |
| **ICD – Auricular (Q17.0/18.1)** | NA | NA | NA | 0.04 | 0.00, 0.19 | **0.002** |
| **ICD – Swelling, Mass, Lump in Neck (R22.1)** | NA | NA | NA | 0.87 | 0.32, 2.02 | 0.765 |
| **ICD – Other (Q18.2)** | NA | NA | NA | 0.63 | 0.27, 1.31 | 0.249 |

**Table S5.** Multivariate logistic regression of prophylactic intravenous antibiotic continuation postoperatively.
