# Supplementary material for: Prophylactic Intravenous Antibiotic Use in Thyroglossal Duct and Branchial Cleft Cyst Excision: A NSQIP‐P Analysis
Source: Otolaryngol Head Neck Surg. 2026 Mar 3;174(5):1243–52. doi: 10.1002/ohn.70186 (PMC13126438; doi:10.1002/ohn.70186)
Supplement: Supplementary file 5 — Supp_TableS6‐8.docx. [file OHN-174-1243-s004.docx]

|  | **Thyroglossal Duct Cyst** | | | **Branchial Cleft Cyst** | | |
| --- | --- | --- | --- | --- | --- | --- |
| **Characteristic** | **OR** | **95% CI** | **p-value** | **OR** | **95% CI** | **p-value** |
| **Postoperative Continuation** | 1.07 | 0.41, 2.43 | 0.887 | 0.43 | 0.02, 2.23 | 0.423 |
| **Antibiotic Class – Penicillin** | 1.53 | 0.35, 4.61 | 0.505 | 0.00 | 0.00, Inf | 0.987 |
| **Antibiotic Class – Clindamycin** | 1.02 | 0.16, 3.53 | 0.983 | 3.86 | 1.10, 10.6 | **0.017** |
| **Time of Administration – Within 5 Minutes Prior** | 0.59 | 0.19, 2.03 | 0.373 | 0.67 | 0.20, 2.58 | 0.525 |
| **Time of Administration – 5-10 Minutes Prior** | 0.79 | 0.29, 2.52 | 0.662 | 1.02 | 0.35, 3.72 | 0.972 |
| **Time of Administration – 10-15 Minutes Prior** | 0.39 | 0.12, 1.34 | 0.112 | 1.21 | 0.40, 4.47 | 0.747 |
| **Time of Administration – 15-30 Minutes Prior** | 0.59 | 0.19, 2.06 | 0.384 | 0.87 | 0.25, 3.42 | 0.827 |
| **Time of Administration – 30+ Minutes Prior** | 0.00 | 0.00, Inf | 0.993 | 0.00 | 0.00, Inf | 0.987 |

**Table S6.** Multivariate logistic regression of postoperative wound infection among patients who received prophylactic intraoperative antibiotics.

|  | **Thyroglossal Duct Cyst** | | | **Branchial Cleft Cyst** | | |
| --- | --- | --- | --- | --- | --- | --- |
| **Characteristic** | **OR** | **95% CI** | **p-value** | **OR** | **95% CI** | **p-value** |
| **Postoperative Continuation** | 1.87 | 0.58, 5.13 | 0.255 | 0.00 | 0.00, Inf | 0.997 |
| **Antibiotic Class – Penicillin** | 0.00 | 0.00, Inf | 0.993 | 0.00 | 0.00, Inf | 0.998 |
| **Antibiotic Class – Clindamycin** | 0.91 | 0.05, 4.65 | 0.925 | 5.69 | 0.27, 44.6 | 0.142 |
| **Time of Administration – Within 5 Minutes Prior** | 1.02 | 0.13, 21.0 | 0.986 | 0.29 | 0.03, 2.71 | 0.251 |
| **Time of Administration – 5-10 Minutes Prior** | 1.57 | 0.26, 30.0 | 0.678 | 0.39 | 0.06, 3.24 | 0.329 |
| **Time of Administration – 10-15 Minutes Prior** | 1.69 | 0.28, 32.4 | 0.629 | 0.17 | 0.01, 1.97 | 0.166 |
| **Time of Administration – 15-30 Minutes Prior** | 3.74 | 0.64, 71.0 | 0.224 | 0.00 | 0.00, Inf | 0.994 |
| **Time of Administration – 30+ Minutes Prior** | 0.00 | 0.00, Inf | 0.996 | 0.00 | 0.00, Inf | 0.998 |

**Table S7.** Multivariate logistic regression of readmission within 30 days among patients who received prophylactic intraoperative antibiotics.

|  | **Thyroglossal Duct Cyst** | | | **Branchial Cleft Cyst** | | |
| --- | --- | --- | --- | --- | --- | --- |
| **Characteristic** | **OR** | **95% CI** | **p-value** | **OR** | **95% CI** | **p-value** |
| **Postoperative Continuation** | 0.71 | 0.20, 2.01 | 0.559 | 0.00 | 0.00, Inf | 0.998 |
| **Antibiotic Class – Penicillin** | 2.38 | 0.53, 7.60 | 0.187 | 0.00 | 0.00, Inf | 0.999 |
| **Antibiotic Class – Clindamycin** | 0.93 | 0.05, 4.70 | 0.945 | 0.00 | 0.00, Inf | 0.998 |
| **Time of Administration – Within 5 Minutes Prior** | 2.37 | 0.38, 45.8 | 0.435 | 0.24 | 0.03, 1.57 | 0.136 |
| **Time of Administration – 5-10 Minutes Prior** | 1.34 | 0.22, 26.0 | 0.790 | 0.19 | 0.02, 1.26 | 0.085 |
| **Time of Administration – 10-15 Minutes Prior** | 1.92 | 0.33, 36.6 | 0.546 | 0.24 | 0.03, 1.59 | 0.137 |
| **Time of Administration – 15-30 Minutes Prior** | 3.90 | 0.69, 73.6 | 0.207 | 0.00 | 0.00, Inf | 0.996 |
| **Time of Administration – 30+ Minutes Prior** | 0.00 | 0.00, Inf | 0.996 | 0.00 | 0.00, Inf | 0.999 |

**Table S8.** Multivariate logistic regression of reoperation within 30 days among patients who received prophylactic intraoperative antibiotics.
